# Book review of “Lanthanide metal-organic frameworks”

**DOI:** 10.3389/fchem.2015.00050

**Published:** 2015-08-11

**Authors:** Victor Borovkov

**Affiliations:** Department of Applied Chemistry, Osaka UniversitySuita, Japan

**Keywords:** book reviews as topic, lanthanides, metal-organic framework, properties, applications

Metal-organic frameworks (MOFs) are chemical systems comprising organic molecules coordinated to metal ions or clusters to form one-, two-, or three-dimensional structural arrangement (Figure 1). The use of lanthanide (Ln) ions results in a specific class of Ln-MOFs possessing unique physico-chemical properties owing to a large atomic magnetic moment, strong spin-orbital coupling, high coordination number, and abundant coordination modes of the Ln ions. Therefore, this research field attracted much attention of a scientific community, hence generating a vast number of research papers and reviews devoted to Ln-MOFs. However, lack of a book addressing exclusively the Ln-MOF systems and covering comprehensively all the major aspects of this research field considerably hinders popularization of this frontier multidisciplinary science as for a general scientific audience and for researchers specializing in other research areas related to MOFs.

**Figure 1:**
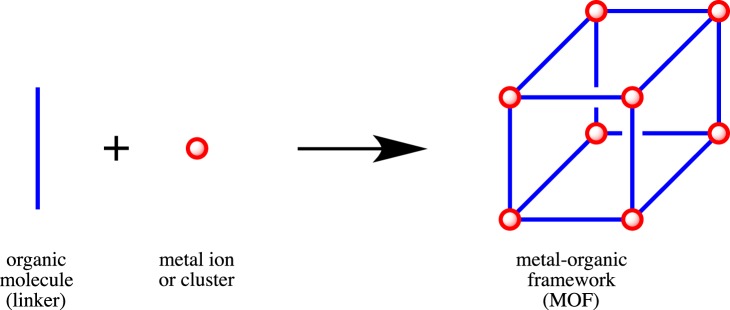
**A general scheme of the three-dimensional metal-organic framework (MOF) construction**.

In connection with this, “Lanthanide Metal-Organic Framework” edited by Peng Cheng fills this gap timely to give an excellent overview of the Ln-MOF systems hence covering the structural diversity, preparation methods, physico-chemical properties, and various applications. In particular, this book consists of nine chapters written by well-known specialists and focuses on all major research topics for Ln-MOFs including synthetic routes for homonuclear, heteronuclear, and nanoscaled complexes, various properties such as porosity, magnetism, chirality, luminescence, host–guest chemistry, and most prospective applications in the fields of gas storage/separation, catalysis, chemical sensors, contrast agents for magnetic resonance imaging, etc. Among these fields the chapters devoted to chiral Ln-MOFs and nanoscaled systems are of particular interest due to its superior novelty and prospecting innovative applications.

All parts of the book are well written and easy for understanding even for a non-specialized chemical readership. However, the book itself is a bit chaotically arranged with some repetitions in different chapters; for example upon discussing some particular properties (such as luminescence, magnetic properties) and applications (such as gas storage, catalysis, sensors), which is a typical weak point of multi-authored book. Also inclusion of the chapter devoted to actinides makes the title of book somewhat misleading, whilst the chapter itself is certainly a valuable addition to a general concept of this book. Despite of these minor shortcomings “Lanthanide metal-organic frameworks” is an outstanding book as for general chemistry readership including graduate and post-graduate students and for researchers specializing in the field of MOF and Ln-MOF. This book can lay foundation for a successful course of further investigations and applications of Ln-MOF and its purchase is highly recommended for everybody who is interesting in this subject.

## Conflict of interest statement

The author declares that the research was conducted in the absence of any commercial or financial relationships that could be construed as a potential conflict of interest.

